# Insights from bauhaus innovation for education and workplaces in a post-pandemic world

**DOI:** 10.1007/s10798-022-09729-2

**Published:** 2022-03-03

**Authors:** Lorraine White-Hancock

**Affiliations:** Faculty of Education, Monash University, Melbourne, VIC Australia

**Keywords:** Bauhaus, Design, Innovation, Transdisciplinary, Transgression

## Abstract

This paper examines Bauhaus School (1919–1933) innovation and relevance today. The School is a landmark in the history of design as a discipline and the development of design education. The School was also a workplace, commercialising Bauhaus-designed products. While drawing global interest in its innovations, the School faced resistance in Germany because it challenged conventions. This problem raises the questions: How did the School-workplace generate innovations amid the calamity of post-war Germany, and what is the significance of the Bauhaus for post-pandemic education and workplaces one hundred years on? The concept of ‘transgression’ is used to understand innovation at the Bauhaus School-workplace. Haraway discusses transgressive practices that disrupt established knowledges, moving ways of thinking and doing in new directions. Analysis of workplace learning research reveals that three interfacing dimensions make up innovation: (1) workplace environments, (2) the culture-order that facilitates innovation, and (3) learning in practice in authentic settings. This qualitative case study reports on how Bauhaus innovation emerged at the intersection of these key dimensions. There are surprising commonalities between the Bauhaus approach to innovation in challenging times and contemporary thinking about supporting innovation which are relevant to education, particularly STEAM (Science, Technology, Engineering, Arts and Mathematics) education, and workplaces in a world affected by the COVID 19 pandemic. Thus, encouraging people who challenge boundaries, rules or ‘the way things are’ can support innovation. This paper addresses a gap in workplace learning research on interrelated dimensions of innovation which the Bauhaus recognized. The study also offers an innovative approach to the examination of innovation across time and space whereas most contemporary studies of innovation focus on the present. Further, conceptualizing innovation as transgression offers a new way of thinking about innovation in design and in the workplace.

## Introduction

The current pandemic has driven the need to reconceptualize the workplace and education. Current conditions have highlighted the need to find innovative ways of addressing challenges to our wellbeing as work and education are disrupted worldwide. Globally, governments have seen innovation as key to wellbeing and economic prosperity but there have been diverse viewpoints about how to generate innovation in workplaces. In contemporary literature on innovation and workplace learning, the role of managers is seen to be critical to enabling or constraining innovation (e.g. Ellström & Ellström, 2018). Some theorists argue that innovation needs to be managed from the ‘top down’ while others argue that innovation is better supported by flat management structures, driven from the ‘bottom up’ by employees (Høyrup, 2012). A ‘safe’ environment is also argued to be needed for learning and innovation to occur (Berthoin Antal & Strauß, 2013). On the other hand, disruption and chaos are seen as positive factors supporting innovation within organizations (Høyrup, 2012). The interaction of physical, social, and mental spaces is also seen to contribute to a workplace environment that either supports or constrains innovation (Heiskanen & Heiskanen, 2011). This paper builds on these debates about what supports innovation in organizations by investigating innovation and the case of the German Bauhaus School (1919–1933).

The Bauhaus School was deliberately designed to foster innovation by crossing boundaries, including boundaries between school and work and disciplinary boundaries. The School faced ongoing criticism because its innovative approaches and activities were seen to be transgressive (Bayer et al., 1938). Yet transgressive practice disrupts established knowledge and can generate new knowledge (Haraway, 2008). Despite resistance, the Bauhaus continued to innovate and spread its influence globally throughout the century (Droste, 2019).

The paper has six further parts. Part One draws on the research literature to understand innovation, transgression and workplace learning. Three key interrelated dimensions of innovation emerged from the literature review: workplaces environments, the workplace culture-order that facilitates innovation and learning in authentic settings. Part Two introduces the German Bauhaus School as a case of innovation. Part Three describes the qualitative, case study research methodology used to investigate innovation and the Bauhaus. Part Four discusses the Bauhaus case in relation to the three dimensions of innovation identified previously. Part Five reports the findings of the study. Part Six draws conclusions about the Bauhaus School’s approaches to innovation which are relevant to workplaces and education today. This part also discusses the significance of transgression for innovation and presents implications of this study for contemporary education, particularly STEAM education.

## Innovation, transgression and workplace learning

### Innovation and transgression

There are multiple meanings attributed to the word ‘innovation’ that largely depend on the context of its use. Generic dictionary definitions tend to relate to newness, change, products, methods and processes. In business and government policy, innovation is generally seen either as an economic process that focuses on product development to generate value or profit or, less often, as a human process that involves new patterns of thinking and acting (White-Hancock, 2017). By contrast, innovation is also defined in terms of social impacts, where the innovation improves people’s lives in some way. The improvement may be aesthetic and emotional rather than just economic (White-Hancock, 2017).

The idea of ‘transgression’ is associated with shifting or movement across boundaries (Haraway, 2008). This movement becomes visible as transgression relative to particular social norms and identities. Foucault (1973) considers movement across boundaries and transgression in terms of power-knowledge relations that are adhered to or rejected as subjected identities resist or challenge established institutional regimes, traditions, systems and truths. Challenges generally come from “foreign elements”, interventions from outsiders (Foucault, 1973, p. 51). Hence, the cultural ‘order of things’ is disturbed when boundaries are transgressed. This disturbance can be viewed as a destructive action that breaches boundaries or limits, such as those imposed by moral codes (Florence, 1998).

Transgression can also be a positive action necessary for change (Foucault, 1973). Transgression involves learning and the evolution of ideas. In the field of education, hooks^1^(sic) (1994) considers transgression as crucial to transformative learning. After all, learning is not fixed and involves movement as learning is gained through experience across time, which can lead to transformation and innovation (White-Hancock, 2018). Writing in the philosophy of science tradition and with a background in evolutionary biology, Haraway (2008) identifies transgression as a positive action that is crucial to evolution. According to Haraway (2008), transgressive symbiotic boundary-crossing processes bring about change in evolutionary development due to the permeability of boundaries:

I love the fact that human genomes can be found in only about 10 percent of all the cells that occupy the mundane space I call my body; the other 90 percent of the cells are filled with the genomes of bacteria, fungi, protists, and such, some of which play in a symphony necessary to my being alive at all (pp. 3-4).

This fluid notion of boundaries creates challenges because the idea of indistinct boundaries transgresses long established and sacred ‘truths’ about ‘humanity’ such as the concept of ‘I’ as a human being that is separate from other life forms. The transgression of boundaries offers opportunities for innovation which Haraway (1991) has long celebrated in terms of “potent fusions”, that is, connections that have the potential to create new ideas (p. 154). These different views of the impact of transgressing sacred boundaries suggest that though boundaries can be crucial sites of innovation through potent fusion, there may be significant challenges for transgressive cross-boundary activity.

### Innovation and workplace learning

Innovation and workplace learning go ‘hand-in-hand’. From a workplace learning perspective, ‘working’ is a form of learning. Knowledge and skills can be developed, shared, adapted, and transformed in workplaces sometimes to the point that formal learning and qualifications are irrelevant to the workplace context (Felstead et al., 2009). Thus, the learning potential within everyday workplace practices (aka, learning in practice) supports innovation.

Recent research attempts to understand the workplace conditions necessary to influence learning that enhances innovation. These studies focus on three key aspects of workplaces relating to ‘authenticity’.

First, participating in authentic workplace settings and in everyday practices is seen to be crucial to learning. Until the early 2000s, there was little empirical research on the complexities, supporting conditions and processes involved in learning in everyday workplace practices or learning in practice that enhances innovation. Since then, studies have investigated occupations within industries (Fenwick, 2004), learning and innovation through regions of economic activity (Felstead et al., 2009), and different work contexts within organizations and across organizational boundaries (Guile, 2009). Guile (2009) has conducted research specifically on learning in the creative industries in England. He identifies ‘new spaces for learning’ as innovative transitional programs from school to work in the creative arts industry as a means of better preparing people for work by learning in practice, that is, direct experience in authentic workplace settings. The experience gained from these programs enabled graduates to learn about norms and conventions in workplaces. Guile’s (2009) study shows how disciplinary and experiential knowledges combine to address the need to learn new ‘rules of engagement’ in developing capacities for short-term project work, self-employment and entrepreneurialism. Hence, learning in authentic settings and in everyday workplace practices is a key component of innovation, particularly in the current industrial landscape of entrepreneurialism.

Second, the culture of the workplace referred to as ‘culture-­orders’ impact on learning and innovation. Workplace or organizational ‘culture’ is defined as “what we do in organizations which inculcates employees with certain cultural values, discourses, emotions, traditions and meanings of artefacts through a process of practice-based learning” (Hasse & Brandi, 2012, p. 135). Until the 2000s, there was an assumption in much organizational studies literature that people will happily engage with innovation and it will flourish if the conditions in the workplace and the innovation system are well managed or controlled from the top down (White-Hancock, 2017). However, reduced funding (due to external events such as pandemics) or profit driven economic imperatives for outcomes and products can affect the willingness of managers to divest control and take risks, tolerate failure or allow time for experimentation, particularly in smaller organizations (Callan, 2004; Curtin et al., 2011). Paradoxically, these aspects of work organization have long been identified with innovation enabling cultures (Callan, 2004). Managers can help construct a supportive workplace culture of learning and innovation by encouraging these enabling factors. On the other hand, ‘employee-driven innovation’ foregrounds innovation driven by employees laterally across an organization, or from the bottom up and through work practice reconstruction. Employees who work collaboratively to share and build knowledge are identified as an overlooked driver of innovation in organizations (Høyrup, 2012). In addition, innovation is seen to be a collaborative top-down/bottom-up process (Holmquist & Johansson, 2019). However there has been little research on which models and processes may be most useful to innovation in times of crisis, such as during the current COVID 19 pandemic.

Third, there are diverse views about what constitutes a supportive workplace environment that encourages innovation. The experience of unexpected and complex situations, disruption, change, new problems and chaos are seen to be characteristic of environments that support or force, innovative learning (Høyrup, 2012). The rapid development of vaccines provides a prime example of the innovative medical response hastened by the pandemic. Encountering blockages or boundaries can also lead people to find innovative ways of solving problems (Felstead et al., 2009). Yet in their review of 268 publications on arts interventions in organizations, Berthoin Antal and Strauß (2013) conclude that a ‘safe’ environment is needed for learning and innovation to occur (Berthoin Antal & Strauß, 2013). A safe workplace is seen to be one that supports employee’s risk-taking because it can be risky to present ideas or work that are different (Berthoin Antal & Strauß, 2013). Hence, seemingly contradictory arguments are presented that the potential for innovation occurs within both safe and chaotic workplaces.

Furthermore, the interaction of physical, social, and mental spaces is seen to contribute to a workplace environment that either supports or constrains innovation (Heiskanen & Heiskanen, 2011). ‘Space’ is defined as a “network of relationships, which creates conditions or environments for human action and interaction” (Heiskanen & Heiskanen, 2011, p. 2). The authors define ‘Physical’ spaces as material facilities, artefacts, and regulatory structures such as “budgets, electronic domains and work schedules” (p. 3). Social spaces refer to social relationships and mental spaces refer to “the world of theory and meanings” (p. 3). The argument is that physical, social and mental spaces work together in a holistic way to create the conditions for human action and interaction, which can either support or constrain innovation in organizations. A consideration of spaces also means talking about boundaries because they locate spaces (Heiskanen & Heiskanen, 2011). Physical spaces have boundaries that are often quite visible. In contrast, social and mental boundaries are often invisible and only become visible when they are transgressed (Heiskanen & Heiskanen, 2011). Therefore, navigating the landscape of these interacting spaces and their boundaries is complex.

This examination of literature found three interrelated dimensions that influence innovation: (1) workplace environments, (2) the workplace culture-order and (3) learning in authentic settings. Thus, there is a need to understand the complexities of workplace learning that enhance innovation (Felstead et al., 2009). An example of where the three dimensions coalesce was the German Bauhaus School.

## The Bauhaus School

The German Bauhaus School was a school of design operational from 1919 to 1933 and was selected for investigation as a case of innovation in the arts and education for four reasons. First, the School was established at a time of economic and social upheaval in post-war Germany (Bayer et al., 1938) as the Spanish Flu pandemic affected populations globally. Rapid change and the need for new approaches and solutions demanded new conditions for creative effort that could contribute to rebuilding the nation (Bayer et al., 1938, p. 21). Comparisons may be drawn across time and space between the Bauhaus era and the world today. The current pandemic has caused similar disruption and turmoil as the conditions in post-war Germany. New approaches and solutions have been needed to address problems created or exacerbated by current conditions. For example, driven by the pandemic is the need to rethink the workplace to include increased networks of home-based, digitally connected spaces. The pandemic has also highlighted the need to reimagine education as inequities of access and digital connectivity are emphasized (Guterres, 2021). Furthermore, creative and collaborative effort is seen to be needed to develop design solutions to emerging problems related to the pandemic (World Design Organisation website, 2021).

Second, the School reimagined the link between education and the world of work. The Bauhaus can be considered a workplace as well as a design school in the sense that students and teachers worked externally in ‘authentic’ settings within industry to manufacture their designs commercially. The School’s new approach recognizes that learning through working in ‘authentic’ settings is as important as theoretical school-based learning (Felstead et al., 2009). Examining the approach of the School shows how learning in authentic industry-based workplaces required and enabled innovation. The dual aims of the Bauhaus were (1) to create a new guild of craftsmen that broke down barriers between artists and craftspeople and (2) to reintegrate the artist into a technological society (Bayer et al., 1938). This reintegration of the arts, crafts and industry was key to generating new knowledge and innovations across workplace and education boundaries.

Third, the Bauhaus is a landmark in the history of design as a discipline, and in the development of design education that draws together different fields of knowledge (Naylor, 1985). The curriculum and the pedagogical approach of the Bauhaus drew together previously separate fields of arts, crafts, business, mathematics, engineering and industry. Subsequently, members of the School produced innovative products that were underpinned by ideas of integration and holism, the organization of work and learning at the School was also highly innovative. However, the Bauhaus program met with criticism and resistance, in part, because this approach challenged the established order of academic and vocational distinction between the disciplines (Bayer et al., 1938). Yet the holistic ideas of the Bauhaus model current integrative STEAM approaches to education which draw together science, technology, engineering, arts and mathematics disciplines.

Fourth, learning emerged at the Bauhaus through cross-boundary practices that transgressed established norms and cultural orders (Bayer et al., 1938). At the School, a work organization and an environment were created to support people who were expected to engage in risky, transgressive work and to innovate. Hence, for these reasons the Bauhaus is a unique case of innovation in the arts and in education.

## Methodology

Learning and innovation at the Bauhaus School was explored through a case study involving historical analysis (Bryman, 2004). This case study is an ‘exemplifying case’ (Bryman, 2004) because it should provide insight into the theories on workplace learning and innovation. This historical case enables the identification of transgressive knowledge practices that generate arts-centred innovation, and rests on assessments of the effects and judgments after the event. The approach makes it possible to see how transgressive knowledge practices emerged and prompted dissent and debate because they challenged conventions*.*

The data sources were the literature on the Bauhaus. Thousands of articles and books have been written about the School. The search was narrowed by sourcing data from the Bauhaus-Archiv: Museum für Gestaltung website written by individuals with first-hand knowledge of the Bauhaus (28 July 2019). The data sources include those directly obtained from the museum website as well as documents listed on the publications page of the website. Four documents used from the website include those written or translated into English. The *Bauhaus Journal* was published between 1926 and 1931 and edited by several Bauhaus teachers. The facsimile edition of all fourteen issues of the journal was used in this study (Müller, 2019). Texts written by László Moholy-Nagy (1950) and Paul Klee (1925/1968) describe the philosophies and pedagogical approaches of these key Bauhaus teachers. *The Bauhaus: 1919–1928* (1938) was co-authored by Walter Gropius, the first Bauhaus Director and Founder; Ise Gropius, whose roles included publicity, public relations and administration at the School and was Walter’s wife; and Herbert Bayer, a key Bauhaus teacher. There were also contributions to this book from eleven other Bauhaus teachers. While not exhaustive, collectively, these documents provided first-hand accounts of the Bauhaus.

There were two key secondary sources. *Bauhaus 1919–1933* authored by Bauhaus expert Magdalena Droste (2019), was located on the on the publications page of the Bauhaus-Archiv: Museum für Gestaltung website (25 March, 2020). The other data source was the literature gathered from web-based searches (19 September 2020). Three other texts were located and selected for review because they were written about the little-known influence of the Bauhaus on art, design and education in Australia and New Zealand (Abel, 2006; Goad et al., 2019; Stasny, 1999). This literature was sourced in several ways. First, an ERIC database search using the key words ‘Bauhaus’ + ‘Australia’ yielded several education texts including Stasny (1999). Second, the search term, ‘Bauhaus influence’ + ‘Australia’, located reference to Goad et al. (2019). Third, a Google search of the Australian architect Harry Seidler, who had been taught by Bauhaus teachers, located the article by Abel (2006).

A critical analysis of primary and secondary documents on the Bauhaus was based on references to the three dimensions found to influence innovation in the literature reviewed: the environment, culture-order, and learning in authentic settings. Salient text was extracted and colour-coded according to these three categories. Colour-coded text was then organized into a table with the three categories, one for each dimension found to influence innovation in contemporary literature reviewed. Key quotes and emerging themes were identified within these categories to draw out patterns, similarities and differences between contemporary research on innovation and workplace learning and the Bauhaus approach, consistent with an historical analysis.

## Dimensions of innovation in the Bauhaus

The next three sections report on how the Bauhaus addressed the three key dimensions of innovation: workplaces environments, the workplace culture-order and learning in authentic workplace settings.

### Workplace environments

Discussion of the conditions in which the Bauhaus artists worked is organized around Heiskanen and Heiskanen’s (2011) ideas about the mental, physical, and ‘social’ spaces that contribute to an environment of innovation in workplaces.

#### Mental spaces

The Bauhaus did not just emerge from the ether. Well before the School opened, the idea of Bauhaus was developing as discourses of collaboration and cultural integration and exclusion were circulating in late 19th and early twentieth century Europe, particularly through the English Arts and Crafts Movement, Russian Constructivism and the Deutsche Werkbund (Naylor, 1985). The German Werkbund, established in 1907, sought co-operation between artists, craftsmen, trade and industry (Bayer et al., 1938). The architect and founder of the Bauhaus, Walter Gropius, had been one of the Werkbund leaders. The concept of uniting the arts and crafts was foreshadowed by William Morris and unfolded with the Arts and Crafts Movement in the 1880s but Morris was not interested in technology or mass production (Bayer et al., 1938, p. 10).

On the other hand, Russian Constructivists saw technology as “the reality of our century” (Moholy-Nagy, 1950, p. 19). Constructivist images of industrial landscapes or sculptural objects were often constructed from metal and scrap machine parts. Influenced by the socialist perspective of the Constructivists, László Moholy-Nagy (soon-to-be Bauhaus teacher) declared, “there is no tradition in technology, no class-consciousness … Constructivism is not confined to the picture frame, and the pedestal. It expands into industry and architecture, objects and relationships. Constructivism is the socialism of vision” (Moholy-Nagy, 1950, pp. 19–21).

Similarly, Werkbund members wanted to integrate the ‘machine style’ of architecture glorifying technology with ideas of the Arts and Crafts Movement (Bayer et al., 1938). This machine style of architecture had been developed in the USA by architects such as Wright and Sullivan and in Europe by van der Velde and Loos. Ideas of unity were also rooted in the medieval guilds of Europe where artists shared the role of craftsmen (Bayer et al., 1938). However, the group was unable to integrate the disciplines on a practical level, and hence, architects, painters and craftsmen still operated as individualists working in their studios in the Romantic tradition (Bayer et al., 1938).

Gropius put the Werkbund theories into operation in a practical way by first amalgamating (in 1919) the Weimar Art Academy and the Weimar School of Arts and Crafts, where he had been the Director (Bayer et al., 1938). This amalgamation reflected the first principle of the Bauhaus Manifesto: “the intellectual, manual and technical training of men and women of creative talent for all kinds of creative work, especially building” (Bayer et al., 1938, p. 98).

The principles of the Bauhaus were addressed through a range of organizational mechanisms and resources which helped to create a ‘safe’ though challenging space for expansive learning and innovation to occur. One of the key mechanisms was the curriculum which has three key features. First, the Bauhaus curriculum was designed to encourage integration and boundary-crossing across previously discreet disciplinary fields. Alongside classes in art theory were studies of materials, tools and technologies, construction techniques, geometry and mathematics and business which provided a broad curriculum for preparing artists to work in an industrial world. The curriculum design reveals the underlying principle of breaking down barriers between the arts and crafts, technology and sciences. This gestalt principle of integration bears kinship with current STEAM approaches to integrated curriculum in education (Radziwill et al., 2015).

Second, the execution of practical, experimental work was a key Bauhaus principle, indicating its importance, and forming the critical foundation of the curriculum from the preliminary course on (Bayer et al., 1938). The approach encouraged work that pushed boundaries. Third, the curriculum incorporated a structure to facilitate collaboration with manufacturing firms. Teachers, students, business managers of the Bauhaus Corporation, factory technicians and manufacturers were all brought into collaborative projects. Income generated from sales of objects produced was divided between the Bauhaus Corporation and the School, which paid the teacher or student who designed the sold object. Thus, the Bauhaus curriculum promoted students’ learning in authentic manufacturing settings and rewarded them for it. Rewarding individuals for their efforts is now seen to be a characteristic of innovative organizations (Callan, 2004).

In summary, principles of integration reflected in the curriculum provided a holistic approach to learning in theory and practice. Transdisciplinary learning, encouraging practical, experimental work, collaboration and learning in authentic manufacturing settings as well as school were key aspects of the curriculum that encouraged innovation.

#### Physical spaces

Physical resources such as the design of the School in Dessau also promoted integration, collaboration and unity, contributing in significant ways to the holistic approach of the Bauhaus. Architectural space was understood to be fundamental to creating an environment that was conducive to expansive learning and innovation at the School. Bauhaus’ means structure (bau) house (haus). The Bau-haus is described as “the collective work of art – the building – in which no barriers exist between the structural and the decorative arts” (Bayer et al., 1938, p. 23). Recent research in the fields of design, architecture and the built environment, and in education, has also been concerned with spatial arrangements that influence learning. Studies have focused on planning physical spaces for different kinds of users and work activities, including creative collaboration (Storvang & Strømgaard Dalby, 2015), on how physical spaces can enable or restrict interaction and creativity (Kristensen, 2004) and interdisciplinary work (Luck, 2014). Recent research also indicates how architectural space can be used to generate change by encouraging interaction and fostering new relationships in organizations (Storvang & Strømgaard Dalby, 2015). The past decade has also seen the emergence of new learning spaces in schools (particularly in the USA, Europe and Australia) which aim to bridge disciplinary boundaries and encourage interaction. ‘Maker spaces’ are designed to support collaborative effort in problem solving as students design and construct objects (Hatch, 2013; Peppler et al., 2016). As students design and construct, they also experiment with materials and techniques, apply scientific and mathematical principles at a practical level, integrating all STEAM disciplines.

Gropius’ design for the Bauhaus at Dessau created an environment that supported interaction, boundary crossing and openness through a range of physical bridging mechanisms. The reinforced concrete ‘skeleton’ of the buildings supported steel window sashes and large double-glazed windows. These were climate controlled ‘curtain walls’ that allowed light to enter the workshops, opening and bridging barriers between interior and exterior, man-made and natural environments. The ‘bridge’ (administration offices), as Gropius called the space, was situated at the intersection of the auditorium, stage and dining hall on one side and the technical school wing, exhibition rooms, halls and laboratory-workshops on the other (Bayer et al., 1938). The term ‘laboratory-workshop’ was also a bridging mechanism that spanned disciplines, symbolically connecting the arts, crafts and sciences with the world of work, and emphasising the experimental nature of these spaces.

At Dessau, a theatre was incorporated into the School and a theatre workshop added to the curriculum to put into practice the concept of *Gesamtkunstwerk *– the total work of art – where collaborative projects were undertaken. The theatre workshop supported the principle of “common citizenship of all forms of creative work, and their logical interdependence on one another in the modern world” (Bayer et al., 1938, p. 125). The theatre brought together many workshops and arts practices necessary for creating a show from architecture, set, graphic and costume design, to music and performance (Bayer et al., 1938). It is an example of an in-between space where new ideas flow and intersect, travelling between and linking different disciplines and workshops. The theatre and workshop reflect current ideas of interactive and collective learning across domains (Felstead et al., 2009). In Harawayan terms, physical and spatial boundaries were treated as porous in the theatre. Thus, the physical spaces at the Bauhaus reinforced the curriculum.

#### Social spaces

The theatre and stage, the dining hall and canteen all accommodated many ‘extra-curricular’ activities and can be considered ‘social’ spaces. These spaces supported the sense of community at the School. Social spaces and the extra-curricular activities taking place in them were also integral to the School’s holistic approach to learning. The Bauhaus community was broadened with invited guests. The School established links with international scholars who were regularly invited there to speak (Müller, 2019). The ‘Circle of Friends of the Bauhaus’ included influential artists (Chagall, Kokoschka), musicians (Schoenberg), architects (Behrens) and scientists (Einstein). These activities reflect ideas of openness and open access that are now seen to optimise innovation (Curtin et al., 2011). Beyond the official curriculum, extra-curricular social activities created an environment that encouraged openness, experimentation and collaboration which encouraged expansive learning in practice.

Interest in the link between work, learning and play is evident in the writing of the influential Bauhaus teacher, Johannes Itten, who proclaimed “play becomes party – party becomes work – work becomes play” (Droste, 2019, p. 79). Concerts, weekly dances, ‘Bauhaus evenings’ and parties were regularly organized by students and teachers. There were lantern parties and theme parties such as the ‘Beard, Nose and Heart Party’ and the ‘Metallic Party’. Almost all workshops such as the furniture/joinery, wall painting/mural, textile/weaving and colour workshops were involved in implementing the broader gatherings (Bayer et al., 1938). The graphic printing workshop also produced postcards and lithographs to advertise events (Droste, 2019). The parties promoted the development of interdisciplinary interaction (Bayer et al., 1938). Oskar Schlemmer who directed the stage workshop, planned and used the parties as a kind of experimental stage for his workshop (Bayer et al., 1938). Indeed, these activities and the spaces where they were performed contributed to innovation in an environment where the freedom to experiment with new forms and risk-taking were applauded (literally). These ‘in-between’ spaces where people were working and not working at the same time, also contributed to a great deal of ‘informal’ learning (Høyrup, 2012). A century ago, Bauhaus members understood how social spaces supported interdisciplinary, interactive and collaborative learning in less formal ways than traditional schooling. Thus, social spaces received as much attention at the Bauhaus as the mental and physical spaces. Similarly, contemporary STEAM learning is supported by new learning spaces that support interdisciplinary curricula, as well as social approaches to learning through collaboration and interaction (Videla et al., 2021).

### The workplace culture-order

Beyond breaking down boundaries between school based, work based and informal learning, the Bauhaus challenged traditional management practices (Bayer et al., 1938). Within the School, a reduced hierarchy of management was established. Bauhaus members developed a collaborative top-down/bottom-up approach which better modelled ideals of unity. Hence, the organization of the School was frequently revised because of student-faculty debate and collaborative decision making (Bayer et al., 1938). The opportunity to participate provided an ‘esprit de corps’ or a ‘shared object’ (to use Felstead and colleagues’ term, 2009) between teachers and students. This approach helped to develop an environment of trust and respect for different ideas to the extent that students referred to themselves as ‘Bauhaus collaborators’ (Bayer et al., 1938). These supports empowered students and staff to help generated change, reflecting recent theories of employee-driven innovation and ‘top-down, bottom-up’ organizational management (for example Høyrup, 2012). These decision-making and governing practices were significant in making internal boundaries more permeable between staff and students and establishing a novel culture within the School. For example, when Gropius resigned in 1928, the newly appointed Director, Hannes Meyer, noted that “the future of the Bauhaus became the subject of violent internal dispute” (Droste, 2019, p. 350). Debate among staff and students lasting several weeks led to reform of the School’s internal structure and curriculum (Droste, 2019). This collaborative decision-making approach challenged the conventional top-down hierarchy of control found within many organizations.

Established knowledge and disciplinary boundaries were also challenged. However, breaking with the traditions of a discipline was found to be challenging for some students. Before Moholy-Nagy took over teaching in the metal workshop at Dessau, it had been a gold and silversmithing workshop making traditional handmade objects–wine jugs, samovars, jewellery and coffee services using traditional precious materials. Moholy-Nagy wanted to address social needs using contemporary, industrial manufacturing techniques, technologies and materials such as nickel, ferrous metals and chromium plating for making models for light fittings and electric household appliances. However, Moholy-Nagy lamented that changing the direction of the workshop “involved a revolution” because the shift in approach challenged the identities of people who saw themselves as craftspeople rather than designer-makers for mass-production (Moholy-Nagy in Bayer et al., 1938, p. 134). Hence, transgressing disciplinary boundaries also meant challenging people’s identities which was no simple process.

Beyond the School, at regional and national levels, Bauhaus ideas and approaches were seen to be radically socialist and transgressive of normative conventions and orderings. Talk of “art-Bolshevism” that “had to be eradicated” started in the media as early as 1919 (Bayer et al., 1938, p. 9). The School was subjected to continual attack by some parts of the media (Bayer et al., 1938, p. 90). Headlines in the papers talk about the “art war”, the “menace” and the “culture demolition” in Weimar, pointing to the controversy that engulfed the School almost from its inception (Bayer et al., 1938, p. 90). Much criticism of the Bauhaus came from members of the academies and the bourgeoisie who the academies supplied with traditional art and architecture (Bayer et al., 1938). Portraits, historical images and decorative landscapes in the grand Romantic style were popular at the time (Bayer et al., 1938). However, Gropius argued that the Romantic ideal of the genius of the individual artist, supported by training in the traditional academies, isolated the artist from the community (Bayer et al., 1938). Furthermore, the academic and vocational distinctions that had developed over the century prior to the Bauhaus, encouraged by the State, were not considered by Gropius to be appropriate to the modern world (Bayer et al., 1938). From this perspective, power structures influenced, shaped and limited knowledge production from the traditional ‘top-down’ approach.

The establishment of the School was hastened by the economic and social turmoil of post-war Germany, and the need for change (Bayer et al., 1938). Yet opposition the School forced two relocations from Weimar to Dessau in 1925 then to Berlin in 1932. Explaining this paradox, Gropius reflected that the confusion of the post-war period exposed a divided Germany of extreme factions where anything new was interpreted as “a sign of some ideological program” (Bayer et al., 1938, p. 11). In Gropius’ view, this resistance to the new explained the force of the attack on the School (Bayer et al., 1938, p. 11). Yet while the School was still active, the “new spirit” demanded new conditions for creative effort that could productively contribute to the nation (Bayer et al., 1938, p. 21). Nevertheless, ongoing political opposition to the principles and activities of the Bauhaus precipitated the closure of the school in Germany in 1933 due to Nazi Party pressure (Bayer et al., 1938).

### Learning in authentic workplace settings

Despite ongoing resistance, new conditions for creative work considered to be more appropriate to the modern world were forged at the Bauhaus. Collaborative learning processes were encouraged to support transgressive practices that cut across established norms. Students were *expected* to become the architects in building bridges to fill the gaps between contemporary ideas about art and existing manufacturing and engineering processes and practices (Bayer et al., 1938). To help achieve this aim, students were required to conduct practical research, to experiment and test the possibilities of materials in order to break away from their traditional use (Bayer et al., 1938). This pedagogical approach contrasts with more traditional approaches where students learned and applied established methods and manufacturing techniques. Teacher then Head of the preliminary course (1928–1933), Josef Albers, argued that this traditional approach “developed discernment and skill, but hardly creative potentialities” (Bayer et al., 1938, p. 114). Yet, under Bauhaus conditions, students built the confidence necessary to make proactive judgements to develop innovations (Albers in Bayer et al., 1938).

Bauhaus students also learned that boundaries can be dissolved, illustrating Haraway’s (2008) argument that boundaries between things are less distinct than we think. Students were taught to look at the structures that lie beneath the surface of things, to examine and visually analyse their geometries through drawing. For Klee (1968) “the art student was to be more than a refined camera, trained to record the surface of the object” (p. 9). Thus, Bauhaus pedagogy focused on developing skills of analysis and practical experimentation, testing and crossing boundaries, similar to the focus of contemporary STEAM education (Videla et al., 2021).

**Fig. 1:**
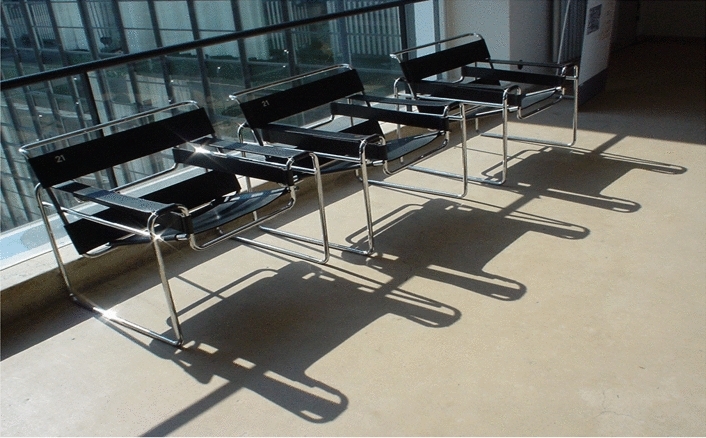
The B3 (*Wassily*) chair. Reproduction, originally designed by Marcel Breuer, 1925–6. Originally called the B3 chair. Photographed by Lorkan, available at: https://commons.wikimedia. org/wiki/File:Bauhaus_3_Chair.jpg under a Creative Commons Attribution 2.0 Generic license

How Bauhaus members applied this analytical, practical and cross-boundary design approach is demonstrated by artefacts which act as lenses for understanding learning through working at the Bauhaus (Felstead et al., 2009). Marcel Breuer’s (1925–6) B3 chair, also known as the Wassily chair (Fig. 1), typifies the Bauhaus design aesthetic arrived at by analysing the essential features and underlying structures of objects, stripping away surface decoration characteristic of objects typically produced at the time by members of the Arts and Crafts Movement. The chair is stripped down to the most basic elements necessary to fulfil its function. Rather than being handmade (like an Arts and Crafts chair), Breuer’s chair was produced with less expensive industrial processes and materials. The tubular steel chair was inspired by bicycle handlebars, reflecting his contemporary, mechanical, industrial world (Furniture webpage, Bauhaus-Archiv: Museum für Gestaltung website, n.d.). In these ways, the artefact responds to Bauhaus theories about artists working across communities to address the needs of everyday life amid the economic and social challenges of post-war Germany.

Bauhaus students learning through working in authentic settings with manufacturers demonstrates contemporary strategies for transitional learning between school and work (Guile, 2009). Director Gropius argued that the school should be “absorbed into the workshop” (Bayer et al., 1938, p. 172). However, this idea is not unique or new to the Bauhaus and occurred elsewhere in Europe. The ‘factory school’ established by Robert Owens in Scotland (1814) and the ‘production school’ first established in France during the eighteenth century share some similar aims (Meyser, 2002).

Visits to factories enabled students to learn about conventional and new manufacturing techniques, processes, equipment and materials. This approach enabled students to design with an understanding of the possibilities and limitations of current manufacturing and to break away from traditional use. The approach expanded learning about the whole design-to-production process and helped to improve design manufacturability (Bayer et al., 1938). In the past, design plans were simply sent to manufacturers (Bayer et al., 1938). The Bauhaus rejected the conventional approach as an inadequate form of communication between designer and manufacturer (Bayer et al., 1938). Students learned not only about modern manufacturing but also new ways of communicating for collaborative work across the school-work divide that were seen to equip them better for a modern, industrialized world.

In summary, Bauhaus artefacts such as the Wassily (B3) chair illustrate how innovations were developed by challenging boundaries such as between art and industry. Innovation was also triggered by the challenges that arose at the boundary between old and new, in times of rapid change and turmoil and through cross-boundary work. In the process of this cross-boundary work, new, hybrid identities were forged at the Bauhaus that did not conform to traditional disciplines (White-Hancock, 2018). This approach was not always a simple or smooth process; the example of the ‘revolution’ in the metal workshop noted earlier shows how Moholy-Nagy’s new industrial focus challenged traditional craft-oriented identities. However, the new direction was supported by the organization, environment and culture of the School.

## Bauhaus influence

While the School’s activities were eventually restricted in Germany due to political resistance, that was not the end of Bauhaus influence (Droste, 2019). Bauhaus ideas and methodology, including those relating to the organization, culture and environment that supported creativity and innovation, spread through its objects and participants. Bauhaus artefacts were exhibited globally. Innovative artefacts embodying Bauhaus ideas acted as powerful tools for mediating knowledge as they were mobilized across networks and boundaries of time and space beyond the School in Germany and throughout the world. Some Bauhaus objects such as the Wassily chair (or very similar versions) are still reproduced which seems surprising in a world that demands the new.

Bauhaus influence also spread as teachers and graduates opened design and architecture practices and taught in prestigious universities around the world, shaping the development of design education (Huang et al., 2020). Bauhaus members emigrated to countries including the USA, Russia, Switzerland, Israel and Australia. Moholy-Nagy and other Bauhaus teachers established the New Bauhaus in Chicago, USA (1937). Breuer taught at Harvard. Gropius was appointed Chair of the Department of Architecture, Harvard Graduate School of Design. Albers became Chairman of the Department of Design at Yale. The prominent Australian architect Harry Seidler attended Harvard Graduate School of Design under Gropius, was Marcel Breuer’s first assistant, and was also taught art by Joseph Albers (Abel, 2006; Goad et al., 2019). Bauhaus member, Ludwig Hirschfeld-Mack, taught art at Geelong Church of England Grammar School in Victoria, Australia during the 1940s and 1950s and is recognized for his influence on art education in Australia (Stasny, 1999). The impact of the Bauhaus on Australian art and education is also demonstrated by the recent staging of the exhibition, *Bauhaus Now* at the Museum of Brisbane, Australia (18 September 2020–18 April 2021) (Bauhaus Now: art+design+architecture: Museum of Queensland). A new Bauhaus Museum is under construction in Germany and the School has been the subject of considerable research interest as extensive celebrations and activities in Germany marked the centenary of the opening of the School (Bauhaus-Archiv: Museum für Gestaltung website). These influential activities, appointments and exhibitions suggest that many cultures saw, and still see, the Bauhaus as a leader of innovation rather than a degenerate organization responsible for ‘culture demolition’ in Germany (Bayer et al., 1938). The movement of Bauhaus ideas and methodology across time and space challenges the idea of the School’s closure in Germany, moving beyond or transgressing the boundary put before it.

## Innovation then and now

Examining the Bauhaus reveals striking parallels with contemporary thinking about innovation. The effects of learning through working on innovation at the Bauhaus rested on the ways in which the Bauhaus addressed three interacting dimensions: workplaces environments; learning in authentic settings; and the workplace culture-order that authorizes innovation. First, the School considered the complex interplay of physical, social and mental spaces which together created the conditions that supported an environment conducive to innovation, as do contemporary researchers such as Heiskanen and Heiskanen (2011). The workplace environment developed enabled students and teachers to challenge and transgress boundaries in order to encourage innovation. The curriculum, pedagogical methods and ‘extra-curricular’ activities were crucial in developing skills and dispositions that supported transgressive activity and innovation. From the preliminary year of the course, questioning and challenging established ways of thinking and doing were nurtured. Critical, analytical and creative thinking, experimentation, risk-taking, communication and collaboration were also encouraged. The skills and dispositions identified as supporting innovation in this Bauhaus study are also identified in contemporary literature on workplace learning and innovation (e.g. Curtin et al., 2011). Such skills and dispositions are also encouraged in current STEAM education (Videla et al., 2021). However, there are also divergent views about what kind of environment constitutes a workplace that encourages innovation. Some researchers conclude that a ‘safe’ workplace environment is necessary (Berthoin Antal & Strauß, 2013). Other researchers suggest that conflict, chaos and disruption lead to expansive learning and innovation (Høyrup, 2012). Both influences on innovation were evident at the Bauhaus. This case study of the Bauhaus shows that different theories about the environmental conditions that support learning and innovation in workplaces can co-exist and are not mutually exclusive.

Second, examining the Bauhaus also illustrates links between innovative workplaces and workplace learning in authentic settings. The design of the Bauhaus produced a ‘workplace’ where artists learned through working with manufacturing industry in authentic settings that generated innovations. Learning about current manufacturing technologies and processes, and the norms and conventions of work practices was seen to be critical for people who were expected to design artefacts using industrial processes. Understanding how the system worked from design to production was also seen as crucial to the development of innovative Bauhaus products. Furthermore, collaborative, interdisciplinary practices were developed with external networks beyond manufacturing industry including the ‘Circle of Friends of the Bauhaus’. In this way, the reach of the Bauhaus workplace was extensive in its endeavor to work and learn with and within broader communities. The approach is consistent with contemporary research arguing the importance of more collaborative and holistic learning across domains and that formal (school-based) qualifications alone are inadequate (e.g. Guile, 2009). Exposure to different ways of thinking and doing models contemporary theories of collaborative and interdisciplinary learning that support workplace learning and innovation (Felstead et al., 2009) and STEAM education today.

Third, a culture-order was developed within the Bauhaus to address perceived ‘new world’ conditions for creative effort that could contribute to the nation. The culture of student-staff collaboration points to a management structure that was relatively flat, also seen to support innovation in contemporary workplaces (Callan, 2004). The School’s organization and culture established a safe space for students to meet challenges and to take risks that led to innovation. This approach was guided by principles of integration and holism, modelling contemporary STEAM education. At the same time, the School sought to meet and address problems not only within it, but also in the broader chaotic culture of the times. In these ways, the Bauhaus was both a social and an aesthetic enterprise. While resistance to new Bauhaus approaches also resulted, this resistance did not crush the ideas of the Bauhaus. Commenting on what the Bauhaus means to him one hundred years on, Mohsen Mostafavi, former Dean of the Harvard Graduate School of Design (HGSD) (2008–2019) which Gropius had led, highlighted the shared focus of the Bauhaus and HGSD on collaboration and transdisciplinary practices:

The Bauhaus was deeply involved in the search for a mode of collaborative pedagogy and knowledge construction, deeply aware of the benefits of engaging in dialogue as well as sharing intellectual common ground … I have never lost sight of the importance of the original vision of the school [HGSD] as a place for collaboration across the disciplines … our focus has remained on the production of forms of knowledge that have the potential not only to enhance a specific field or discipline but also to play a productive role in the spatial and social transformation of the built environment. To achieve this goal has necessitated an understanding of the relationship between disciplinary knowledge and transdisciplinary practices... (Mostafavi, 2019).

Thus, consistencies between Bauhaus and contemporary design education are evident. This paper also points to many consistencies between the School’s approach to innovation and recent research on the conditions conducive to workplace learning and innovation. These consistencies across time and space highlight the relevance of the Bauhaus to education, particularly STEAM education, and workplace innovation now.

## Concluding comments

Investigating innovation and the Bauhaus illuminates the complexities of learning that supports innovation in workplaces. This investigation helps to clarify how the interrelationships between the three dimensions of innovation — the workplace environment, learning in authentic settings, the culture-order are implicated in innovation. Thus, workplaces and managers can better support and build capacity for innovation by recognizing how the three key dimensions together can enable innovation.

This Bauhaus study also illuminates the relationship between innovation and transgression. Transgressions at the boundaries - between school (knowledge) and work (practice), organizational and disciplinary boundaries — enabled Bauhaus innovation. The Bauhaus illustrates Haraway’s (2008) ideas about boundaries that are culturally embedded and ordered but are not impenetrable and can be transgressed or moved ‘through’. Contemporary research tends to investigate innovation in workplaces in the ‘here and now’ and often does not provide a detailed picture of the developments or effects of transgressions and their relation to ‘culture-orders’, which makes innovation visible. This Bauhaus study uses the benefits of hindsight to grasp the character of transgression and the terms and conditions that produce transgressive knowledge practices and innovation.

The School’s approach required and enabled transformation as boundaries were transgressed and different ways of doing and knowing were encountered and integrated. Collaboration and transdisciplinary work were critical to knowledge production, transformation and innovation at the Bauhaus. This transformative movement involved epistemological and ontological shifts that challenged identities and transgressed ‘sacred’ boundaries. In the process of transgressive, cross-boundary work, new, hybrid identities were forged that did not conform to traditional disciplines, and a new discipline was born. The implication for contemporary education systems and organizations is that transgression can be a positive action and an indicator of innovation that manifests when boundaries become a *conduit* for interaction and communication, rather than division. Hence, though it may be challenging, encouraging people who contest boundaries, rules or ‘the way things are’ in organizations and education systems can support innovation.

This study argues the need to reconceptualize education, the workplace and the links between them as means of encouraging innovation. The study indicates how holistic and transdisciplinary approaches to learning such as those found in emerging STEAM education can encourage innovation. STEAM education can play a productive role in preparing young people to address post-pandemic problems and there is value in expanding STEAM curricula. Finally, the study argues that learning in authentic workplace settings and beyond schools is a key component of innovation. However, questions are raised about the impact of decreased opportunities for learning in authentic workplace settings as a consequence of lockdowns in the current pandemic. There is need for further research about what this impact means for learning and innovation in occupations and workplaces. There is also need for research on innovative new ways of working and new work spaces emerging amid conditions generated by the current pandemic.

## Footnotes

1 The author uses lower case letters for her name because she believes that the substance of her writing is what is important rather than who she is (Williams, 2013).
